# Emotion recognition specialization and context-dependent risk of anxiety and depression in adolescents

**DOI:** 10.1002/brb3.299

**Published:** 2015-01-16

**Authors:** Albertine J Oldehinkel, Catharina A Hartman, Floor V A Van Oort, Esther Nederhof

**Affiliations:** 1 Department of Psychiatry, Interdisciplinary Center Psychopathology and Emotion regulation (ICPE), University of Groningen, University Medical Center GroningenGroningen, The Netherlands; 2 Department of Child and Adolescent Psychiatry/Psychology, Erasmus Medical Center, Sophia Children's HospitalRotterdam, The Netherlands

**Keywords:** Anxiety/anxiety disorders, child/adolescent, depression, family/marital, stress

## Abstract

**Background:**

Some adolescents function poorly in apparently benign environments, while others thrive despite hassles and difficulties. The aim of this study was to examine if adolescents with specialized skills in the recognition of either positive or negative emotions have a context-dependent risk of developing an anxiety or depressive disorder during adolescence, depending on exposure to positive or harsh parenting.

**Methods:**

Data came from a large prospective Dutch population study (*N* = 1539). At age 11, perceived parental rejection and emotional warmth were measured by questionnaire, and emotion recognition skills by means of a reaction-time task. Lifetime diagnoses of anxiety and depressive disorders were assessed at about age 19, using a standardized diagnostic interview.

**Results:**

Adolescents who were specialized in the recognition of positive emotions had a relatively high probability to develop an anxiety disorder when exposed to parental rejection (*B*_specialization*rejection_ = 0.23, *P* < 0.01) and a relatively low probability in response to parental emotional warmth (*B*_specialization*warmth_ = −0.24, *P* = 0.01), while the opposite pattern was found for specialists in negative emotions. The effect of parental emotional warmth on depression onset was likewise modified by emotion recognition specialization (*B *=* *−0.13, *P* = 0.03), but the effect of parental rejection was not (*B *=* *0.02, *P* = 0.72). In general, the relative advantage of specialists in negative emotions was restricted to fairly uncommon negative conditions.

**Conclusions:**

Our results suggest that there is no unequivocal relation between parenting behaviors and the probability to develop an anxiety or depressive disorder in adolescence, and that emotion recognition specialization may be a promising way to distinguish between various types of context-dependent reaction patterns.

## Introduction

Adolescents interact with their environment in diverse and complex ways. Supportive environments are generally considered healthier than adverse ones, yet adverse conditions do not affect all youth in the same way. Some adolescents function poorly in apparently benign environments, while others seem to thrive despite hassles and difficulties (Kim-Cohen 2007). This suggests that person-environment (mis)matches, rather than simply good or bad environments, predict developmental outcomes (Nederhof and Schmidt 2012). For children and adolescents, the family environment is a highly important and enduring context (Schor 2003), hence skills that provide the optimal tools to deal with their specific family characteristics (i.e., matching skills) may be particularly potent to promote mental health and prevent psychopathology. This raises the question which skills enable adolescents to adequately cope with their familial context, and so support, in the longer term, their mental health (Moos 2002). The study here described was built on the premise that emotion recognition skills might play such a role, and aimed to examine if adolescents with specialized skills in the recognition of either positive or negative emotions have a context-dependent risk of developing an anxiety or depressive disorder, depending on whether their family environment matched these skills.

Individuals differ in how well they can perceive facial emotions, and these differences are partly emotion-specific (Schlegel et al. 2012). Facial emotion processing has been investigated in relation to many psychiatric disorders, among which anxiety (Heuer et al. 2007) and depression (Leppänen 2006). These disorders are assumed to have their own characteristic emotion processing patterns, (e.g., Joormann and Gotlib 2006; Bediou et al. 2012), for instance, anxiety-prone individuals have been postulated to be particularly fast in recognizing cues signaling threat (Williams et al. 1997; Surcinelli et al. 2006), and depression-prone individuals to be fast in recognizing sadness (Lopez-Duran et al. 2013) and slow in recognizing happiness (Surguladze et al. 2004). However, the available empirical evidence regarding the specific nature of the deviations is characterized by many inconsistencies (Clark and McManus 2002; Lang and Sarmiento 2004; Bistricky et al. 2011; Kohler et al. 2011; Bediou et al. 2012). This might indicate that the balance between how well different emotions are perceived is more relevant than the skill to perceive an individual emotion per se, because the balance is more informative regarding possible attentional biases. It might also indicate that the risk associated with particular emotion recognition skills partly depends on contextual factors.

We hypothesized that specialists in the recognition of either positive or negative emotions have an advantage in family environments that match these skills (i.e., positive and harsh parenting, respectively), and a disadvantage in nonmatching environments, as compared to individuals without specialization. This hypothesis was based on the consideration that controllability and predictability are key contributors to the experience of stress (Koolhaas et al. 2011), a major contributor to a wide range of (mental) health problems. Rather than a stimulus itself, the degree to which it is perceived as controllable and predictable determines whether or not it has negative health consequences (Salvador 2005; Weiss 2008). In case of frequent exposure to harsh parenting, recognition of negative emotions may help to predict and control the environment, and so prevent or escape harm. Conversely, when there is no need to be prepared for parental harshness, openness to positive emotions may facilitate better use of favorable parenting circumstances (Kerckhoff 2007; Ellis et al. 2011; Hankin et al. 2011). The other side of the coin is that individuals who specialized in the recognition of particular emotions are disadvantaged in family environments in which these emotions are relatively rare. We investigated the consequences of (mis)matches between emotion recognition specialization and parenting conditions with regard to adolescent onsets of anxiety and depressive disorders in a large prospective population study of adolescents.

## Materials and Methods

### Sample and procedure

The data were collected as part of the TRacking Adolescents' Individual Lives Survey (TRAILS), a prospective cohort study of Dutch adolescents with bi- or triennial measurements from age 11 onwards (Huisman et al. 2008; Ormel et al. 2012). The present study was based on data from the first (T1, 2001–2002) and fourth (T4, 2008–2010) wave. At T1, 2230 (pre)adolescents were enrolled in the study (response rate 76%, mean age 11.1, SD = 0.6, 51% girls; De Winter et al. 2005), of whom over 83% (*N *=* *1881, mean age 19.1, SD = 0.6, 52% girls) participated again at T4 (Nederhof et al. 2012). At T1, the participants filled out questionnaires at school, supervised by one or more test assistants. In addition, the school assessments involved individual sessions, including the measurement of emotion recognition (see below). The T4 assessment wave comprised a psychiatric diagnostic interview, which was completed by 84% of all T4 participants (*N* = 1584), of whom 1539 (97%, 841 girls, 698 boys) had valid data on all variables used in the present study. The study was approved by the Dutch Central Committee on Research Involving Human Subjects. Participants were treated in compliance with the Declaration of Helsinki, and all measurements were carried out with their adequate understanding and written consent.

### Measures

#### Emotion recognition

Emotion recognition skills were assessed at T1, by means of a reaction-time (RT) task of the Amsterdam Neuropsychological Task program (ANT; De Sonneville 1999). Participants were tested individually, by trained undergraduate psychologists. The ANT started with a task in which the participants had to push a button as soon as a square was presented on the screen, to assess a baseline speed measure. The emotion recognition task consisted of six parts of each 20 target and 20 nontarget trials. Each part lasted about 5 min and focused on a particular emotion, that is, happiness, sadness, anger, fear, disgust, and surprise. The participants had to push a yes-button if the face presented on the computer screen matched the target emotion and a no-button if the face shown had another expression. Reaction times more than four standard deviations above the mean (Stevens 2002) as well as participants performing at chance level of accuracy (50% or more errors) were considered missing.

For each emotion, we calculated the mean RT of the correct answers and divided these RTs by the baseline speed to adjust for differences that were not directly related to emotion recognition skills. The adjusted RT for happiness was used as a measure of recognition of positive emotions, while the adjusted RTs for sadness, anger, fear, and disgust were used as measures of recognition of negative emotions. The mean of the adjusted RT for positive and negative emotions was used as a measure of general Emotion Recognition Time (ERT), with higher scores indicating poorer emotion recognition skills. The difference between the standardized (z) scores for the recognition of negative and positive emotions was calculated as a measure of Emotion Recognition Specialization (ERS), in such a way that a high score represented relatively short RTs for positive emotions (i.e., a specialization in positive emotions) and a low score relatively short RTs for negative emotions (specialization in negative emotions).

#### Perceived parenting

The EMBU (a Swedish acronym for My Memories of Upbringing) for Children (EMBU-C; Main et al. 1985; Markus et al. 2003; Muris et al. 2003) was developed to assess children's perception of parental rearing practices. Each item can be rated as 1 = never, 2 = sometimes, 3 = often or 4 = almost always; and is asked for both the father and the mother. We used the scales Rejection (12 items, Cronbach's *α *= 0.84) and Emotional Warmth (18 items, *α *= 0.91) to assess harsh and positive parenting, respectively. Rejection is characterized by hostility, punishment, derogation, and blaming of the child. Emotional Warmth refers to giving special attention, praising for approved behavior, unconditional love, and being supportive and affectionate. The EMBU-C was administered at T1. Because the answers for both parents were highly correlated (*r *=* *0.67 for Rejection, *r *=* *0.79 for Emotional Warmth), they were averaged into a single measure.

#### Psychiatric disorders

Psychiatric disorders were assessed at T4, using the Composite International Diagnostic Interview (CIDI), version 3.0 (Kessler and Ustun 2004; Haro et al. 2006; Kessler et al. 2009). The CIDI is a structured diagnostic interview, which yields diagnoses according to the criteria of the Diagnostic and Statistical Manual of Mental Disorders (DSM-IV), as well as their age of first onset, which was used to exclude onsets during or prior to the age at T1 (see Analysis). Anxiety Disorder was defined as a diagnosis of Agoraphobia, Generalized Anxiety, Panic Disorder, Separation Anxiety, Social Phobia, or Specific Phobia. Obsessive-Compulsive Disorder and Post-Traumatic Stress Disorder were not included in the category of Anxiety Disorders. There is an ongoing debate on whether or not these disorders should be considered anxiety disorders, and both were classified otherwise in the DSM-5 (Stein et al. 2011, 2014). Depressive Disorder was operationalized as a Major or Minor Depressive Episode or Dysthymia.

#### Psychiatric symptoms

Psychiatric symptoms were assessed at T1 by the Youth Self-Report (YSR; Achenbach 1991). The YSR contains a list of 112 behavioral and emotional problems, which children can rate as 0 = not true, 1 = somewhat or sometimes true, or 2 = very or often true in the past 6 months. We used the Anxiety Problems scale (six items, Cronbach's *α *= 0.63) and the Affective Problems scale (13 items, *α *= 0.77; Achenbach et al. 2003).

### Analysis

First, we calculated descriptive statistics of the variables used in this study as background information for the interpretation of the results. After that, Cox proportional hazards models were used to estimate main and interaction effects of emotion recognition specialization (ERS), Parental Rejection and Parental Emotional Warmth on the probability to develop an Anxiety or Depressive Disorder (first onset). To avoid reverse causality (i.e., parental rejection or emotional warmth being the consequence of the psychiatric disorder rather than the cause) and heterogeneity due to combining childhood- and adolescent-onset disorders, individuals with ages of onset less than or equal to their age at T1 were excluded from the analyses. Separate models were tested for the two parenting styles and the two outcomes. All analyses were adjusted for gender and overall Emotion Recognition Time (ERT). Continuous variables were standardized to mean 0 and standard deviation 1 prior to analysis. In the first step, the main effects of ERS and parenting were included in the model. The second step involved the inclusion of the interaction of ERS and parenting. In the third step, T1 (i.e., pre-onset) Anxiety or Affective Problems were added to the model to check the direction of the effects. In the fourth step, we additionally included Depressive Disorder (in models with onset of Anxiety Disorder as outcome variable) or Anxiety Disorder (in models with onset of Depressive Disorder as outcome) to assess the extent to which the associations were unique for the outcome disorder or due to comorbidity. Comorbid Anxiety or Depressive Disorder was included as a time-dependent covariate, hence only exerted its influence from its age of onset onwards. Finally, we fitted a model in which both Rejection and Emotional Warmth were included simultaneously to estimate the amount of overlap of their individual effects. In addition to the regression coefficients (*B*), the effects were also expressed in hazard ratios (HRs), which reflect the (instantaneous) relative risk of onset. To illustrate the nature and size of the effects, logistic regression analyses were used to estimate risks of onsets between T1 and T4, conditional on parenting and ERS. All analyses were performed using SPSS version 20.0 (IBM, Armonk, New York). Statistical tests were two-tailed and *P*-values < 0.05 were considered statistically significant.

## Results

### Descriptive statistics

The ERTs, defined as emotion recognition RTs divided by baseline speed, indicated that recognizing a particular emotion took, on average, about three times longer than pressing a button as quickly as possible (Table 1). Within the emotions, happiness was recognized faster (adjusted RT = 2.66, SD = 0.56) than the negative emotions (mean adjusted RTs = 3.42, range 3.22–3.69, SDs 0.69–0.83), and the correlations among negative emotions (mean *r *=* *0.69, range 0.65–0.73) were somewhat higher than the correlations between positive and negative emotions (mean *r *=* *0.60, range 0.57 –0.65). Girls were slightly faster than boys in recognizing emotions (*t* [df = 1537] = 3.41, *P* < 0.01), but the genders did not differ in ERS (*t* = 1.05, *P* = 0.30). At T1, girls reported more Anxiety Problems (*t* = 5.38, *P* < 0.01) than boys and a comparable number of Affective Problems (*t* = 0.92, *P* = 0.36). The lifetime prevalence of Anxiety Disorders was 26.1%, and of Depressive Disorder 21.3%. Of those, disorders that started at an age less than or equal to the age at T1 (Anxiety: *n *=* *264, Depression: *n *=* *53) were excluded from the analyses. During adolescence, 10.8% of the sample that was still at risk developed an incident Anxiety Disorder and 18.4% an incident Depressive Disorder. More specifically, the incidence of Agoraphobia was 0.2%, of Generalized Anxiety 2.2%, of Panic Disorder 0.9%, of Separation Anxiety 1.7%, of Social Phobia 5.5%, and of Specific Phobia 2.0%; while the incidence of a Major Depressive Episode was 14.5%, of a Minor Depressive Episode 1.7%, and of Dysthymia 4.0%. Comorbidity within categories was limited: 1.6% of the sample developed more than one anxiety disorder, and 1.7% more than one depressive disorder. The incidence of both Anxiety and Depressive Disorder was significantly higher in girls than in boys (Anxiety: 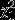 = 16.0, *P* < 0.01; Depression: 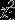 = 40.2, *P* < 0.01).

**Table 1:** Distribution of the variables used in this study

	Mean (SD) or %		
Variables	Total sample *N* = 1539	Girls *n* = 841	Boys *n* = 698
Age at T1	10.8 (0.7)	10.8 (0.7)	10.9 (0.7)
Age at T4	18.7 (0.7)	18.7 (0.7)	18.7 (0.6)
Emotion Recognition Time (ERT)^1^	3.05 (0.57)	3.00 (0.56)	3.10 (0.58)
Emotion Recognition Specialization (ERS)^2^	0.00 (0.78)	−0.02 (0.74)	0.02 (0.84)
Parental Rejection	1.48 (0.31)	1.45 (0.29)	1.52 (0.32)
Parental Emotional Warmth	3.23 (0.49)	3.27 (0.48)	3.18 (0.49)
T1 Anxiety Problems (range 0–2)	0.36 (0.31)	0.40 (0.32)	0.32 (0.29)
T1 Affective Problems (range 0–2)	0.30 (0.25)	0.31 (0.24)	0.29 (0.25)
Onset of Anxiety Disorder after T1 (*n* = 1275)^3^	10.8%	14.1%	7.2%
Onset of Depressive Disorder after T1 (*n* = 1486)^3^	18.4%	24.3%	11.5%

1 Mean reaction times divided by baseline speed. A higher score indicates slower emotion recognition.

2 Difference between the standardized recognition times for positive and negative emotions. A positive score indicates a specialization (i.e., relatively short reaction times) in positive emotions; a negative score indicates a specialization in negative emotions. Please note that ERS was calculated as the difference between the standardized RTs for positive and negative emotions, and therefore has mean zero by definition.

3 Varying sample sizes due to different numbers of adolescents excluded because of onsets before T1.

Parental Rejection was positively associated with T1 Anxiety and Affective Problems, and Emotional Warmth negatively. Emotional Warmth was not associated with onsets of psychiatric disorders during adolescence; Rejection only with Depressive Disorder (Table 2). Emotional Warmth correlated negatively with ERT and positively with ERS. The associations were generally weak.

**Table 2:** Associations among emotion recognition skills, parenting behaviors, and adolescent-onset psychiatric disorders

	I	II	III	IV	V	VI	VII
I. Emotion Recognition Time (ERT)^1^							
II. Emotion Recognition Specialization (ERS)^2^	0.04						
III. Parental Rejection	0.01	−0.01					
IV. Parental Emotional Warmth	−0.07*	0.06*	−0.37*				
V. T1 Anxiety Problems	−0.02	0.02	0.32*	−0.09*			
VI. T1 Affective Problems	0.00	0.02	0.40*	−0.20*	0.62*		
VII. Onset of Anxiety Disorder^3^	−0.05	−0.04	0.04	0.04	0.14*	0.13*	
VIII. Onset of Depressive Disorder^3^	−0.00	−0.04	0.08*	−0.03	0.16*	0.17*	0.17*

Total *N* = 1539; correlations with psychiatric disorders are based on lower numbers due to exclusion of pre-T1 onsets.

* *P* < 0.05.

1 Mean reaction times divided by baseline speed. A higher score indicates slower emotion recognition.

2 Difference between the standardized recognition times for positive and negative emotions. A positive score indicates a specialization (i.e., relatively short reaction times) in positive emotions; a negative score indicates a specialization in negative emotions.

3 The figures reflect point-biserial correlations (with continuous variables) or Phi coefficients (with nominal variables).

### Prediction of psychopathology

Table 3 shows whether and how perceived parenting interacted with ERS in predicting first onsets of Anxiety and Depressive Disorder. The positive interaction of ERS and Parental Rejection and the negative interaction of ERS and Parental Emotional Warmth in the prediction of Anxiety Disorder indicate that adolescents with a negative ERS (i.e., specialized in negative emotions) had a relatively low probability to develop an Anxiety Disorder when exposed to Parental Rejection and a relatively high probability in case of Parental Emotional Warmth, as hypothesized. The interaction effects remained virtually similar after adjusting for comorbid Depression and pre-onset Anxiety Problems. With regard to the onset of a Depressive Disorder, Rejection appeared a potent predictor, but its effect largely disappeared after adjustment for pre-onset Affective Problems. The effect of Rejection was, against expectations, not modified by ERS. Largely in keeping with the hypotheses, Emotional Warmth had a protective effect for adolescents with a positive, but not for those with no or a negative ERS. Again, these effects could not be ascribed to the presence of comorbid Anxiety or pre-onset Affective Problems. When adjusted for each other, the effects of Rejection and Emotional Warmth remained mostly comparable, but the interaction of ERS and Emotional Warmth in the prediction of Anxiety dropped below significance level (*B *=* *−0.12, HR = 0.89, *P* = 0.27).

**Table 3:** Cox regression models for the prediction of an onset of anxiety and depression during adolescence

	a and b and c			+d			+e			+f		
	*B*	HR	*P*	*B*	HR	*P*	*B*	HR	*P*	*B*	HR	*P*
Adolescent Onset of Anxiety Disorder (*N *=* *1275)												
a. Emotion Recognition Time (ERT)	−0.16	0.85	0.09	−0.16	0.85	0.08	−0.17	0.85	0.08	−0.18	0.84	0.06
b. Emotion Recognition Specialization (ERS)^1^	−0.09	0.91	0.31	−0.13	0.88	0.18	−0.15	0.86	0.12	−0.16	0.86	0.11
c. Parental rejection	0.18	1.19	0.03	0.14	1.15	0.10	0.03	1.03	0.76	0.01	1.01	0.93
d. ERS^*^Parental Rejection				**0.23**	**1.26**	**<0.01**	**0.23**	**1.26**	**0.01**	**0.25**	**1.28**	**0.01**
e. T1 Anxiety Problems							0.34	1.40	<0.01	0.31	1.36	<0.01
f. Depressive Disorder										0.89	2.43	<0.01

a. Emotion Recognition Time (ERT)	−0.15	0.86	0.11	−0.16	0.85	0.09	−0.16	0.85	0.09	−0.17	0.85	0.08
b. Emotion Recognition Specialization (ERS)^1^	−0.11	0.90	0.25	−0.11	0.90	0.25	−0.13	0.88	0.15	−0.14	0.87	0.16
c. Parental Emotional Warmth	0.10	1.11	0.27	0.08	1.09	0.36	0.12	1.13	0.18	0.13	1.14	0.16
d. ERS^*^Parental Emotional Warmth				**−0.24**	**0.79**	**0.01**	**−0.21**	**0.81**	**0.03**	**−0.20**	**0.82**	**0.04**
e. T1 Anxiety Problems							0.36	1.43	<0.01	0.32	1.38	<0.01
f. Depressive Disorder										0.88	2.40	<0.01

Adolescent Onset of Depressive Disorder (*N *=* *1486)												
a. Emotion Recognition Time (ERT)	0.02	1.02	0.76	0.02	1.02	0.76	0.02	1.02	0.73	0.04	1.04	0.52
b. Emotion Recognition Specialization (ERS)^1^	−0.06	0.94	0.33	−0.06	0.94	0.31	−0.07	0.93	0.28	−0.06	0.94	0.33
c. Parental Rejection	0.23	1.26	<0.01	0.23	1.26	<0.01	0.09	1.09	0.17	0.07	1.08	0.27
d. ERS^*^Parental Rejection				**0.02**	**1.02**	**0.72**	**0.02**	**1.02**	**0.76**	**0.00**	**1.00**	**0.99**
e. T1 Affective Problems							0.32	1.38	<0.01	0.26	1.30	<0.01
f. Anxiety Disorder										0.93	2.53	<0.01

a. Emotion Recognition Time (ERT)	0.02	1.02	0.81	0.01	1.01	0.85	0.02	1.02	0.81	0.03	1.04	0.58
b. Emotion Recognition Specialization (ERS)^1^	−0.05	0.95	0.39	−0.08	0.92	0.20	−0.09	0.92	0.16	−0.08	0.92	0.20
c. Parental Emotional Warmth	−0.12	0.89	0.05	−0.12	0.88	0.04	−0.05	0.96	0.47	−0.05	0.95	0.44
d. ERS^*^Parental Emotional Warmth				**−0.13**	**0.88**	**0.03**	**−0.13**	**0.88**	**0.03**	**−0.12**	**0.89**	**0.04**
e. T1 Affective Problems							0.35	1.42	<0.01	0.28	1.33	<0.01
f. Anxiety Disorder										0.93	2.53	<0.01

All effects were adjusted for gender. *B*, regression coefficient; HR, hazard ratio. Effects related to the main hypotheses of this study are in boldface.

1 Difference between the standardized recognition times for positive and negative emotions. A positive score indicates a specialization (i.e., relatively short reaction times) in positive emotions; a negative score indicates a specialization in negative emotions.

**Figure 1:**
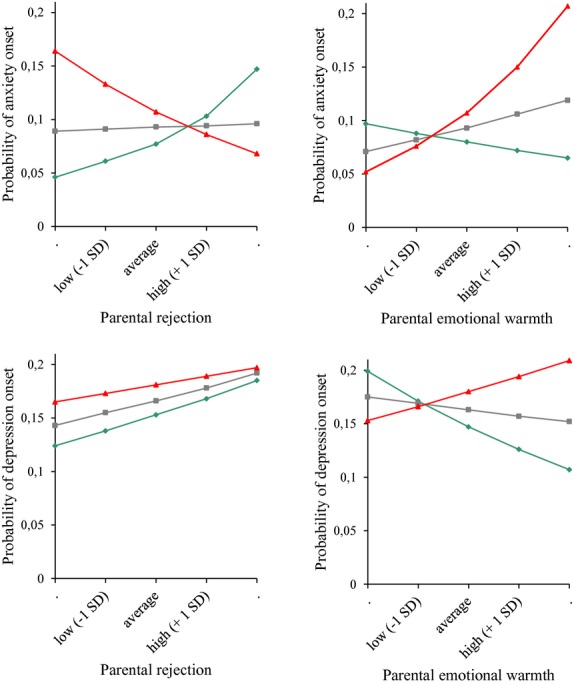
Probability of onset of anxiety and depression between age 11 (T1) and 19 (T4), conditional on perceived parenting and emotion recognition specialization (ERS) at T1. The green lines (diamond markers) represent the estimated risks for adolescents with a positive ERS (+1 SD), the red lines (triangle markers) the risks for adolescents with a negative ERS (−1 SD), and the gray lines (square markers) risks for adolescents without a specialization (ERS = 0). The effects are adjusted for gender, general emotion recognition skills, and, respectively, T1 anxiety and depression symptoms.

A graphical representation of the conditional risk of onsets, based on logistic regression analyses, is given in Figure1. It is interesting to note that the largest differences between the ERS groups were found under relatively favorable rearing circumstances (i.e., low Rejection, high Emotional Warmth).

## Discussion

The aim of this study was to examine if adolescents with specialized skills in the recognition of either positive or negative emotions would have a context-dependent risk of developing an anxiety or depressive disorder, depending on whether their family environment matched these skills. We hypothesized that specialists in the recognition of positive emotions would have an advantage when exposed to positive parenting and a disadvantage when exposed to harsh parenting, compared to individuals without specialization, and expected the opposite pattern in specialists in the recognition of negative emotions. The results partly corroborated these hypotheses; how emotion recognition specialization modified associations between parenting behaviors and psychopathology appeared outcome dependent.

With regard to the development of anxiety disorders, our findings yielded support for the notion that specialists have context-dependent advantages and disadvantages. The relative advantage of specialists in negative emotions appeared restricted to fairly uncommon negative conditions: only then did their estimated chance to develop an anxiety disorder become lower than that of generalists and specialists in positive emotions (Fig.1). This suggests an overall unbalance between the benefits of a positive and a negative emotion recognition specialization, at least in youth from relatively advantaged, secure populations like our sample: in most contexts adolescents with a positive emotion recognition specialization are better off than those with a negative specialization.

The association between perceived parental emotional warmth and depressive disorders was modified by emotion recognition specialization as well: whereas high emotional warmth decreased the probability of depression in adolescents with a positive emotion recognition specialization, it increased depression risk in those with a negative specialization. The effect of parental rejection was not modified by emotion recognition specialization: children exposed to parental rejection had an increased risk for depression regardless of emotion recognition specialization.

The difference between anxiety and depression regarding individual differences in the effect of parental rejection is remarkable. Why the dual nature of specialization was found with regard to anxiety but much less so with regard to depression is as yet a matter of speculation. Possibly, the assumed increased predictability and controllability of the environment in case of matching emotion recognition skills can reduce feelings of threat. Whereas the experience of threat is a core feature of anxiety disorders, its association with depressive disorders is less self-evident and probably indirect at most. Consistent with this, prior research has indicated that an early attention bias for threat, including high vigilance for negative facial emotions, was more strongly associated with anxiety than with depressive disorders (Mogg et al. 2000; Mathews and MacLeod 2005).

Adolescents with a negative emotion recognition specialization were more likely to develop an anxiety or depressive disorder at high levels of emotional warmth than in its absence. One might wonder why parental emotional warmth could induce psychopathology in some adolescents. It is conceivable that high levels of parental emotional warmth provide a home environment that is so comfortable that it reduces adolescents' need to seek fulfillment in the outside world and engage in the adolescent transition from parents to peers (Cyranowski et al. 2000). Consequently, these adolescents may miss opportunities to overcome social fears and thus be more likely to develop a (social) anxiety disorder, particularly if they are specialized in the recognition of negative emotions. Indirect support for this supposition comes from research showing that parental overprotection, which tends to be associated with emotional warmth (Oldehinkel et al. 2006), restricts children's possibilities to develop a sense of mastery (Chorpita and Barlow 1998; Van der Bruggen et al. 2008), and can predispose them to the development of anxiety and depression (e.g., Rapee 1997; Chorpita and Barlow 1998; Oldehinkel et al. 2006; Van der Bruggen et al. 2008; Murray et al. 2009).

Although the main focus of this study was on emotion recognition specialization rather than emotion recognition skills as such, it is worth noting that emotion recognition time was not associated with (risk of) anxiety and depression in this study. This might be due to nonlinear effects; that is, perhaps the probability of mental health problems is high not only in adolescents who are overall poor at emotion recognition, but also in those who can recognize emotions exceptionally well. The former group may be troubled by low perceived environmental controllability and predictability (Salvador 2005; Weiss 2008), the latter by high sensory-processing sensitivity, which has been associated with perceived stress (Benham 2006) and negative affect (Aron and Aron 1997).

In addition, please note that the main effects of rejection and emotional warmth on adolescent-onset anxiety disorders were not significant, while their effects on depression onsets were only significant when unadjusted for pre-onset symptoms. This is consistent with prior reports based on the same sample suggesting that the effect of parental rejection on anxiety symptoms decreases over the course of adolescence (Van Oort et al. 2011; Legerstee et al. 2013). The present study adds to this knowledge that this decrease does not occur in all adolescents.

Our study has a number of notable strengths: a large sample of adolescents, a follow-up period of 8 years, and information about the onset of DSM-IV anxiety and depressive disorders assessed by standardized diagnostic interviews. The combination of these factors offered unique opportunities to investigate context-dependent benefits and risks of emotion recognition specialization prospectively.

Two limitations should be accounted for when interpreting the findings. First, parenting was operationalized as adolescents' perception of upbringing. Because children are influenced by their parents' rearing behavior through their mental representations of this behavior (Main et al. 1985), perceived parenting was considered the most relevant measure, but its drawback is that it leaves much room for subjective interpretations. Report bias was probably limited, though, because several studies have concluded that the impact of mental health on reported parental rearing is minimal (Gotlib et al. 1988; Gerlsma et al. 1991). Moreover, report bias, if any, is unlikely to have affected the interaction effects found. Second, emotion recognition specialization was defined on the basis of a single emotion recognition task, which included more negative than positive emotions and measured speed, rather than accuracy of emotion recognition. These particularities of the Amsterdam Neuropsychological Task program (De Sonneville 1999) may have influenced the reaction times and so our measure of emotion recognition specialization. Replication with other emotion recognition tasks is therefore needed to confirm the findings.

## Conclusions

In sum, our results suggest that there is no unequivocal relation between parenting behaviors and the probability to develop an anxiety or depressive disorder in adolescence, and that emotion recognition specialization may be a promising way to distinguish between various types of context-dependent reaction patterns. Nevertheless, much remains to be learned about the actual conditions under which a specific specialization may be beneficial. Another issue that deserves further study concerns the implications of emotion recognition skills for prevention or intervention strategies; it is conceivable that individuals with a positive and individuals with a negative specialization require different strategies in order to be optimally effective. For example, the association between high emotional warmth and anxiety onset in adolescents specialized in negative emotions could imply that these adolescents function better in (therapeutic) environments characterized by relatively little warmth and empathy. Our study may thus contribute to the further development of evidence-based tailor-made interventions.
